# Echocardiography in the evaluation of athletes

**DOI:** 10.12688/f1000research.6595.1

**Published:** 2015-06-15

**Authors:** Gonzalo Grazioli, Maria Sanz, Silvia Montserrat, Bàrbara Vidal, Marta Sitges

**Affiliations:** 1 Cardiology Department, Hospital Clínic, Universitat de Barcelona, IDIBAPS, Institut d’Investigacions Biomèdiques August Pi iSunyer, Barcelona, Catalonia, Spain

**Keywords:** echocardiography, athletes, pre-participation screening, athlete’s heart

## Abstract

Echocardiography is currently a widely available imaging technique that can provide useful data in the field of sports cardiology particularly in two areas: pre-participation screening and analysis of the cardiac adaptation induced by exercise.

The application of pre-participation screening and especially, the type and number of used diagnostic tests remains controversial. Echocardiography has shown though, higher sensitivity and specificity as compared to the ECG, following a protocol adapted to athletes focused on ruling out the causes of sudden death and the most common disorders in this population. It is still a subject of controversy the actual cost of adding it, but depending on the type of sport, echocardiography might be cost-effective if added in the first line of examination.

Regarding the evaluation of cardiac adaptation to training in athletes,  echocardiography has proved to be useful in the differential diagnosis of diseases that can cause sudden death, analysing both the left ventricle (hypertrophy cardiomyopathy, dilated cardiomyopathy, left ventricle non compaction) and the right ventricle (arrhythmogenic right ventricular cardiomyopathy).

The aim of this paper is to review the current knowledge and the clinical practical implications of it on the field of echocardiography when applied in sport cardiology areas.

## Contributions of echocardiography in athletes

The number of people practicing sport has increased about five fold over the past 30 years ^1^. The benefits of sport practice in improving cardiovascular health are unquestionable ^2^, but an increase in cardiovascular events has also been demonstrated during its practice ^3^. As a consequence, the absolute number of people at risk of sudden cardiac death (SCD) during exercise is also increasing ^4^. Sports activity is not a cause of the increased mortality per se; rather, it might act as a trigger of cardiac arrest in athletes with structural or electrical heart abnormalities that generating malignant arrhythmias. Thus, it seems reasonable that every effort should be made for early recognition of any disease that may put the athlete at risk, keeping in mind the perspective that inadequate disqualification of individuals might also pose a risk.

Therefore, a pre-participation screening (PPS) protocol seems to be of interest. Consequently, the European Society of Cardiology ^5^ has proposed an exam which emphasizes three points or steps: a) family and personal history, b) physical examination and c) 12-lead electrocardiogram (ECG). The ECG has demonstrated a 70% sensitivity to detect the most frequent causes of SCD in young athletes ^6, 7^. However, about a third of these athletes with an anomalous origin of coronary arteries, aortic diseases and incipient forms of cardiomyopathies will present with a normal ECG.

The echocardiogram might be a useful, non-invasive and accessible tool to increase sensitivity of screening ^8^. Our group reported the echocardiographic findings among 2688 competitive athletes; most of the echocardiographic evaluations were normal and only 203 (7.5%) showed abnormalities ^9^. Cessation of athletic activity was indicated in 4 athletes: 2 for hypertrophic cardiomyopathy (electrocardiography had shown changes that did not meet diagnostic criteria), 1 pectus excavatum with compression of the right ventricle, and 1 significant pulmonary valve stenosis; the other minor alterations in echocardiography (7.5% of the total population) did not entail cessation of athletic activity and only indicated periodic monitoring.

Although rare, some cardiac structural changes can be missed on physical examination and electrocardiography; in contrast, they are easily recognized with echocardiography. These findings suggest the use of echocardiography in at least the first PPS of competitive athletes to improve the effectiveness of programs aimed at preventing SCD in athletes.

Currently, there is no consensus on what kind of echo scan has to be included in the PPS in order to detect the most prevalent cardiac abnormalities related to SCD. Some studies have suggested a quick 5 minute echocardiogram protocol ^10^ while other studies have proposed longer protocols performing a complete echocardiogram ^11^. In our group, we carry out the standard transthoracic echocardiographic views suggested by the European Society of Echocardiography ^12^; we consider that the long-axis parasternal, the short-axis parasternal, the apical 4-chamber views and 2-chamber views, suprasternal and parasternal right view provide a high sensitivity to diagnose the most prevalent causes of SCD (summarized in Table 1).

**Table 1.** Echocardiographic scan protocol.

View	Focus in …
LV Parasternal long-axis view (2D + M-mode + colour Doppler)	• LV hypertrophy or dilatation • Aortic and Mitral morphology and function • Aortic root (Marfan) • Ascending aorta (Marfan)
Parasternal short-axis view at aortic valve level (2D + colour Doppler)	• Coronary artery origin • Aortic valve morphology • Pulmonary valve • Atrial septum • Ventricular septum • Persistent Ductus • LV hypertrophy • LV non-compaction
Parasternal right view	• RV morphology and function
Apical 4-chamber view (2D + colour Doppler + CW aortic)	• LV function • RV morphology and function • Aortic, mitral, and tricuspid valves morphology and function • Atrial and ventricular septum • Pulmonary veins
Suprasternal view	• Aortic arch • Arterial Ductus
Subcostal view	• RV morphology and function • Atrial septum • Ventricular septum

The most common abnormalities detected in athlete’s echocardiograms can be divided into two different groups: physiological structural and functional cardiac adaptive changes that result in what is called the athlete’s heart, and echocardiographic signs of different cardiomyopathies that can induce SCD ( Table 2).

**Table 2.** Echocardiographic findings in athletes.

Athlete’s Heart	• Ventricular dilatation and hypertrophy • Atrial dilatation • Ventricular hypertrabeculation • Mild aortic dilatation
Sudden Cardiac Death	Most common: • Hypertrophic cardiomyopathy • Anomalous origin of coronary artery Less common: • Aortic dilatation (Marfan) • Myocarditis • Mitral valve prolapse Uncommon: • Arrhytmogenic RV cardiomiopathy • Atherosclerotic coronary artery diseases • Aortic valve stenosis

Hypertrophic cardiomyopathy constitutes the leading cause of SCD in young athletes ^13^. The ECG has demonstrated a high sensitivity in the diagnosis of this entity, but there is still around 10% of patients with hypertrophic cardiomyopathy, who have abnormal ECGs ^14– 16^. On the other hand, 9% of the athletes with mild adaptive left ventricular (LV) hypertrophy show pathological changes in the ECG ^17^. In both situations an echo scan would help to achieve the correct diagnosis.

The anomalous origin of the coronary arteries was considered to be a rare cause of SCD in athletes, but nowadays it has been demonstrated that it can be related to up to half of the previously asymptomatic SCD cases ^15^. It was the second cause of sudden death associated with sports in the largest register of SCD in athletes ^13^. The resting ECG of these athletes is normal, so this entity, if asymptomatic, cannot be detected in a regular PPS based on anamnesis, physical examination and ECG. It is known that an echocardiogram performed by physicians with adequate training can differentiate coronary anomalies with high sensitivity ^18^, and therefore echo is again a key tool to unmask these asymptomatic patients.

Aortic root diseases are an infrequent cause of SCD in young people ^17^, although they seem to be a more prevalent cause of SCD in athletes ^19^. Echocardiogram allows for the diagnosis as well as the follow up of these patients. Similarly, bicuspid aortic valve without significant functional abnormalities would not be diagnosed in a regular PPS, and again, the echocardiogram would allow for an early diagnosis and a proper follow up ^20^.

Finally, an echo scan is recommended in congenital heart disease patients, with special focus on ventricular morphology and function, pulmonary pressure and aortic diameters before starting an exercise protocol ^21^.

## Usefulness of echocardiography in pre-participation screening

To evaluate the cost effectiveness of the addition of a complementary study to the standard PPS, we have to take into account three basic parameters: a) incidence of SCD related to sport practice in the population b) cost of the study; c) years of potential life saved.

To date, there is no consensus which is the real incidence of SCD related to sports practice, because it depends on the analyzed population. Studies in Italy ^22^ and Israel ^23^ have reported an incidence of 2 cases of sudden death per 100,000 athletes per year, while other studies in France have documented an incidence of < 1 per 100,000 athletes ^4^. On the other hand, the cost to perform an echocardiography is significantly different between American countries ^7^ and Europe ^24^. Finally, the quantification of the years of potential life lost depends on which population is considered, from school age ^25^ or all young adults up to master athletes engaged in sports ^26^. The weight of each of these three factors (incidence of SCD, cost of studies, years of life saved) in a given population to determine the cost-effectiveness of adding echocardiography in PPS represent the challenge that we will have to face in the upcoming years.

Currently, it is still controversial if the inclusion of an ECG in the regular PPS is cost effective ^27^ in the USA, while in Europe this recommendation was established more than 10 years ago ^5^ and it has been adopted by the different sport committees ^28^ and international federations ^29^. Several studies have demonstrated the cost-effectiveness of including an ECG in the PPS ^7, 30^, but to date, only a small population study in school-aged athletes has analyzed the cost-effectiveness of adding an echocardiography in the screening ^31^; the study showed that adding echocardiography increased both the cost and the sensitivity. In our opinion, the echocardiography provides a higher sensitivity of the PPS, especially in some special populations with greater amount of cardiac disorders described such as competitive athletes ^9^, sports with high static component ^32^ or long distance endurance athletes ^33^. Recommendations for PPS and use of echocardiography used by our group according to the level of sport practice and training are summarized in Table 3.

**Table 3.** Cardiovascular pre-screening protocols according to the level of sport practice ^32^. F&P: family and personal, PE: physical exam.

	History F&P + PE	ECG	Echocardiography
Recreational sports	Yes	Yes	No
Competitive athletes, High static and dynamic component Endurance training, > 10 hours/week	Yes	Yes	Yes

## Differentiating physiologic adaptation from pathology

The structural and functional adaptive changes that the heart develops in response to exercise, classically called “athlete’s heart“, has intrigued clinicians and scientists for more than a century. In the 19 ^th^ century, Henschen described for the first time sport induced cardiac enlargement by auscultation and percussion ^34^. Seventy years after the first athlete’s electrocardiographic features were described ^35, 36^ and a few years after the first 2-dimensional echocardiography images showed the characteristic chamber enlargement and myocardial hypertrophy of the athlete’s heart. Finally, the current advanced echocardiography techniques and magnetic resonance imaging (MRI) have begun to clarify the mechanism involved in these athlete’s heart adaptive features. The study of the athlete’s heart is thus essential, not only to understand how cardiac adaptation contributes to improved athletic performance, but also to differentiate the athlete’s heart from important disease states which may share similar morphologic features. We briefly review the physiologic and morphologic features associated with athletic training and the keys to differentiate normal adaptive athlete’s heart features from mild or initial expression forms of cardiac diseases such as hypertrophy cardiomyopathy (HCM), dilated cardiomyopathy (DCM), left ventricle non compaction (LVNC) and arrhythmogenic right ventricular cardiomyopathy (ARVC).

## Factors influencing cardiac remodeling in athletes

Different forms of exercise impose different loads on the cardiovascular system. Classically, two forms have been described according to their hemodynamic effect. Endurance exercise results in an increased cardiac output due to the rise in heart rate and stroke volume, reduced peripheral resistance and moderate increment in systemic blood pressure, leading to a volume overload. On the other hand, strength exercise is characterized by a maintained or a slightly increased cardiac output and peripheral vascular resistance, which results in increased blood pressure and thus an increased LV afterload. Cycling or running are examples of endurance exercise while weightlifting is an example of strength exercise, but there are also overlapped sports combining endurance and strength hemodynamic conditions in different proportions such as soccer or hockey. These different hemodynamic conditions will result in different cardiac adaptive structural and functional changes. Moreover, cardiac remodeling is not a continuous response to exercise; it is influenced by individual genetic factors, gender and race. Thus, a proper athlete’s evaluation should be individual and take into account these potential influencers.

## The left ventricle

Endurance exercise LV remodeling is typically described as LV chamber enlargement with increased wall thickness resulting in an eccentric LV hypertrophy, while strength remodeling is described as a thickening of the LV wall with a slight increase in the size of the LV cavity resulting in a concentric hypertrophy. This dichotomous view, first described by Morganroth *et al.* ^37^ is currently controversial. A meta-analysis by Pluim *et al.* ^38^ initially confirmed this model; in contrast, a recent meta-analysis by Utomi *et al.* ^33^ did not find this classic concentric remodeling in strength athletes and only found a slight LV dilatation and similar LV wall thickness as in endurance athletes. An increase in LV wall thickness is a typical feature of the athlete’s heart, however it is usually minimal and within normal range. In a cohort of 947 elite athletes Pelliccia *et al.* ^39^ found a LV wall thickness > than 13 mm in only 1.7% of the athletes. Sharma *et al.* ^40^ in a cohort of 720 elite athletes also reported a low incidence with only 0.4% of subjects showing LV wall thickness > 12 mm among elite junior athletes. However, this small number of extreme cases of exercise-induced remodeling may be difficult to differentiate from mild forms of hypertrophic cardiomyopathy. Various studies have tried to find out the key to differentiate these two entities, but to date there is no pathognomonic sign available, and a combination of clinical and family history, electrocardiographic and echocardiographic features is recommended. The increase in LV wall thickness in athletes is an adaptation to increase stroke volume so it has to be accompanied by chamber enlargement. Thus, a LV end-diastolic diameter > 54 mm ^41^, an increased LV volume and particularly LV volume/mass ratios by MRI ^42^ have been proposed to differentiate athlete’s heart from disease ^41^.

**Figure 1.**
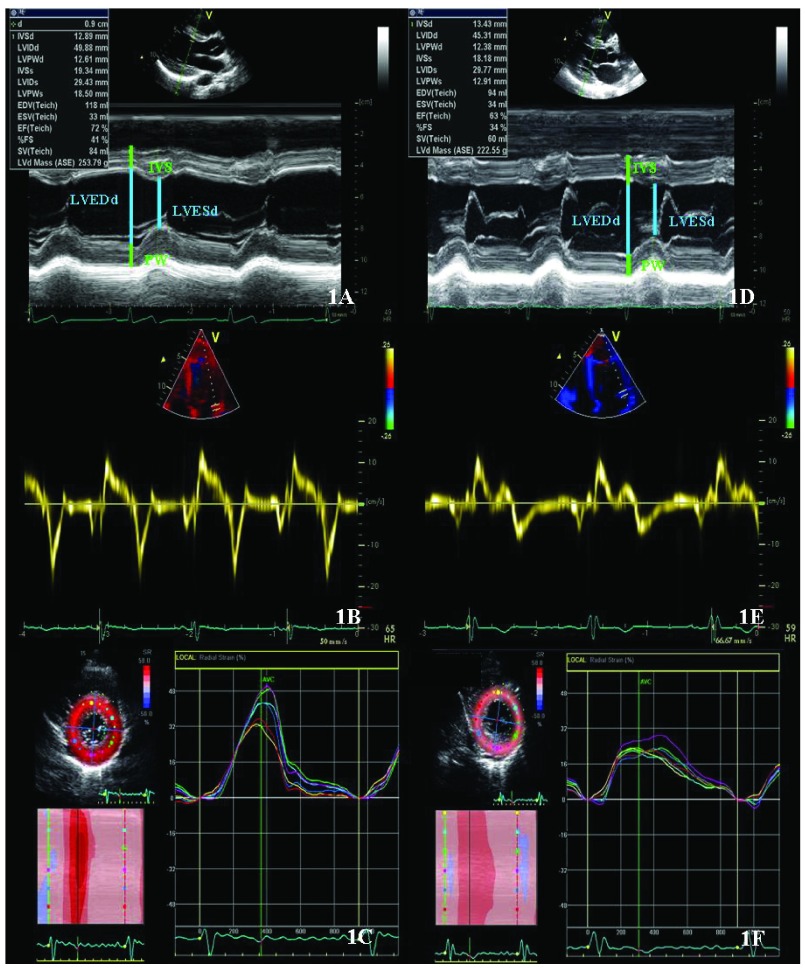
Echocardiographic assessment of an athlete with left ventricular hypertrophic adaptation (Figure 1: A, B, C) and a patient with a mild form of hypertrophic cardiomyopathy (Figure 1: D, E, F). Patients with hypertrophic cardiomyopathy present smaller LV end-diastolic diameters, reduced radial strain values and reduced velocities of the mitral annulus as compared to athletes.

Recent advances in echocardiographic techniques including Tissue Doppler Imaging (TDI) and Speckle Tracking Imaging (STI) permit an accurate assessment of the myocardial function, helping us to differentiate adaptation from disease. Numerous studies have demonstrated normal or even supranormal diastolic LV function in athletes ^43^; instead, the pathological forms of LV hypertrophy are typically associated with an impaired diastolic dysfunction characterized by lower early diastolic mitral annulus velocity ^44^. Thus, the evaluation of diastolic LV function by TDI is nowadays mandatory in the evaluation of LV hypertrophy ^16^. Figure 1 illustrates this echocardiographic differential diagnosis. In cases where differential diagnosis is unclear, MRI is useful. MRI offers a more accurate assessment of LV wall thickness, cardiac volumes, and tissue composition. Furthermore, adding gadolinium for late enhancement, the presence and location of myocardial fibrosis can be determined.

As previously described, LV cavity enlargement is part of the cardiac remodeling observed in response to exercise, but this LV dilatation is in most cases minimal and indexed LV cavity dimensions are below pathologic limits. However, in the selected population of endurance elite-athletes this LV remodeling can be extreme. In a study by Pelliccia *et al.* ^45^ more than 10% of elite ultra-endurance athletes had LV cavity end-diastolic dimensions > 60 mm, simulating forms of DCM. LV systolic function is described to be in a normal range among athletes ^46^, but again, studies including high intensity endurance athletes have revealed a slight LV systolic dysfunction with LV ejection fraction around 45–49% ^47^. In these extreme cases, the new advanced echocardiographic techniques can also help us. Although slightly lower ejection fraction of the LV might be found, the adaptive cardiac remodeling shows normal or even supranormal values of strain and strain rate by TDI, and normal values of longitudinal strain assessed by STI ^48^. In contrast, in DCM patients these values are reduced ^49^. The effect of endurance training on ventricular deformation, torsion and untwisting rate needs further investigation, but promising findings report exercise-induced supranormal LV untwisting rates ^50^, confirming again the physiological LV response to exercise.

Finally, parallel to the improvement in echocardiographic techniques and image resolution, a surprising high prevalence of LV hypertrabeculation has been described in athletes. Gati *et al.* ^51^ in a cohort of 1146 athletes studied by echocardiography, reported trabeculations in 20% of the athletes, and even more, around 8% fulfilled conventional criteria for the diagnosis of LV non-compaction cardiomyopathy; this prevalence raised to 13% when only black athletes were considered. LV non-compaction cardiomyopathy is a rare cardiomyopathy thought to be secondary to the arrest of normal myocardial development, resulting in multiple deep ventricular trabeculations ^52^. This entity has a wide clinical expression from asymptomatic patients to advanced cases with three characteristic symptoms: heart failure, thromboembolic events and fatal arrhythmias ^53^, and has indeed been related to exercise-related SCD in young athletes ^54^. The mechanisms implicated in LV hypertrabeculation in athletes are still unknown, but the reported high prevalence suggests that it might be another expression of cardiac adaptation to increased preload and afterload influenced by genetic and ethnical factors ^51^. Structural echocardiographic features that could help to differentiate cardiac adaptive remodeling from disease are: the location of trabeculations (apical region in LV non-compaction cardiomyopathy versus mid-cavity region in athletes) and the evidence of late enhancement in cardiac MRI following gadolinium in LV non-compaction cardiomyopathy ^54^. Furthermore, LV non-compaction cardiomyopathy patients may have reduced systolic and diastolic function while athletes with LV hypertrabeculation normally have no systolic or diastolic dysfunction. In the few cases with slightly low LV ejection fraction, a normal or even supranormal increase in LV systolic function with exercise could help us to distinguish pathology from physiological adaptation ^51^.

## The right ventricle

**Figure 2.**
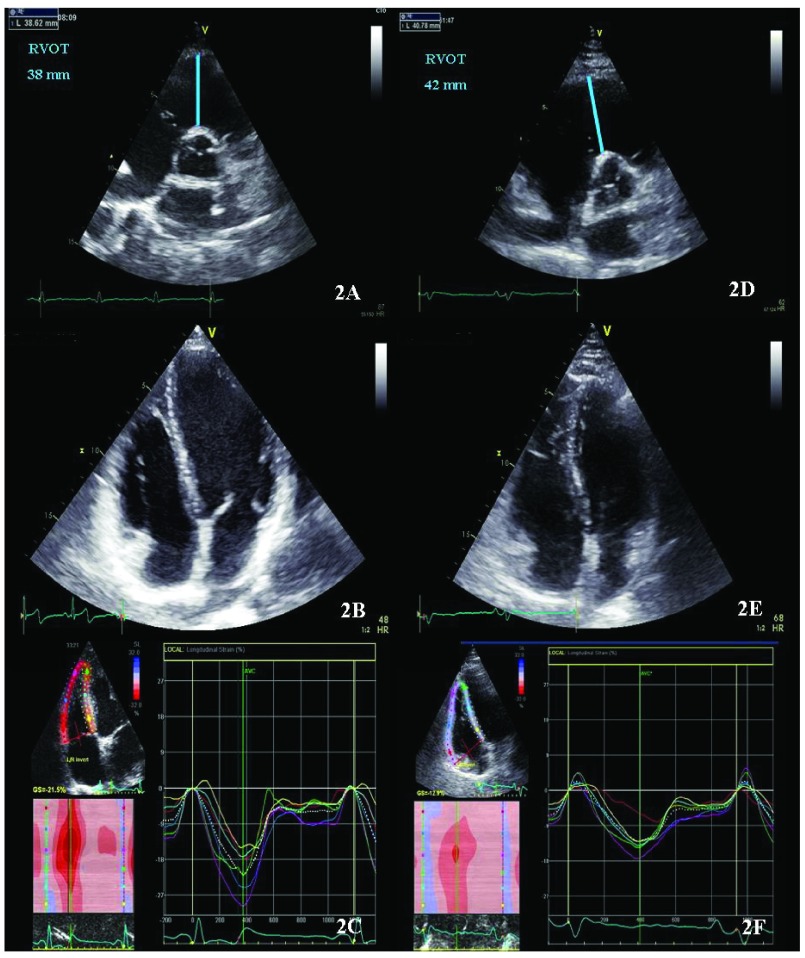
Echocardiographic assessment of an athlete with right ventricle remodelling (Figure 2: A, B, C); and a patient with arrhythmogenic right ventricular dysplasia in early stage (Figure 2: D, E, F). Patients with arrhythmogenic right ventricular dysplasia in early stage can present only mild RV dilatation but the relationship between LV and RV cavity tend to be less than 1 and the RVOT is at least mildly dilated and they present reduced RV global and segmental strain values as compared to athletes.

During exercise, both ventricles have to increase stroke volume in response to the increased cardiac output demanded during exercise. This workload imposes high stress to all myocardial structures, which seems to be especially important in the right ventricle (RV) that typically works at low pressures in physiological conditions ^55^. Classically, the study of the athlete’s heart was focused on the LV; but in the last two decades, with the introduction of advanced echocardiographic techniques and MRI, the RV exercise-remodeling has started to be described. Structurally, endurance exercise has been related to RV enlargement, typically balanced with LV dilatation ^56^. Functionally, high intensity endurance exercise has been related to lower global RV peak systolic longitudinal strain values at rest as compared to controls; the RV basal is the segment most affected in this change ^57^. To date, whether these lower strain values are the result of myocardial damage ^58^ or are only an adaptive response demonstrated by an increased reserve after exercise provocation ^59^ is still controversial. So far, few studies have focused on the RV in strength athletes but such RV remodeling seems to be less pronounced ^60^. Extreme RV remodeling cases in elite ultra-endurance athletes may be indistinguishable from arrhythmogenic right ventricular dysplasia (ARVD). ARVD is a desmosomal cardiomyopathy characterized by progressive adipose and fibrosis myocardial infiltration with potential bad prognosis and constitutes one of the most important causes of sudden death in young athletes ^13^. Various studies have demonstrated more rapid disease progression in patients that practice moderate-high intensity exercise, making the differential diagnosis between disease and cardiac remodeling even more challenging ^61^. As previously mentioned, endurance exercise can induce adaptive physiologic biventricular dilatation, where the ratio of LV/RV remains unchanged. On the other hand, a reduced LV/RV ratio could be a warning sign of underlying disease ^62^. In addition, athlete’s RV remodeling has proved to be global as opposed to that observed in ARVD patients who show a disproportionate enlargement of the RV outflow tract. Functionally, no motion abnormalities have been described in athletes ^62^ despite having a lower deformation in the basal segment of the RV; abnormal motion of the RV is essential data to fulfil the ARVD diagnostic criteria ^63^. Figure 2 illustrates this echocardiographic differential diagnosis. Finally in doubtful cases, MRI can provide us an accurate structural and functional RV evaluation, distinguishing segments with dyskinesia, fibrosis or outflow tract microaneurysms ^64^. However, to date, there is no single sign available to differentiate both entities and consequently, a combination of clinical and family history, electrocardiographic and echocardiographic features are recommended. Table 4 summarizes the main echocardiographic features used to differentiate athlete’s heart from early stages of myocardial disease.

**Table 4.** Echocardiographic features to differentiate athlete’s heart from cardiomyopathies. **LV GLS:** Left ventricle Global Longitudinal Strain.

	Hypertrophy cardiomyopathy	Athlete’s heart
**LV end-diastolic** **diameter**	≤ 45mm	> 54mm
**LV volume/LV mass**	Reduced	Normal
**E´ mitral lateral annulus**	Reduced	Normal or supranormal
**LV radial and** **circumferential Strain**	Decreased	Normal or supranormal
	**LV non compaction** **cardiomiopathy**	**Athlete’s heart**
**Trabeculation location**	Apical	Mid-cavity
**E´mitral lateral annulus**	Normal or reduced	Normal or supranormal
**LV GLS at rest** **LV GLS during effort**	Normal or reduced Reduced	Normal or slightly reduced Normal or supranormal
	**Arrhythmogenic right** **ventricular dysplasia**	**Athlete’s heart**
**RV enlargement**	Early RVOT dilatation	Global
**Motion abnormalities**	Yes	No
**Ratio RV/LV volumes**	≥ 1	< 1

## Conclusions

In summary, echocardiography is a useful imaging tool to detect underlying heart disease that may imply a risk for people practicing sport and at the same time is a non-expensive and non-invasive technique to evaluate cardiac adaptation to training. The challenge remains the diagnosis and differentiation of extreme adaptation to training that very much resembles early stages of some myocardial diseases. Recently developed tools to better quantify cardiac performance have improved this issue but still more knowledge on the pathophysiology of cardiac adaptation to training is needed to optimize the identification of subjects at risk for sudden death or irreversible cardiac damage.
