# Supplementary material for: Echocardiography in the evaluation of athletes
Source: F1000Res. 2015 Jun 15;4:151. [Version 1] doi: 10.12688/f1000research.6595.1 (PMC4516021; doi:10.12688/f1000research.6595.1)
Supplement: Peer review history

# Peer review: Referee response for version 1

**DOI:** 10.5256/f1000research.7082.r9498

**Published:** 2015-07-27

**Authors:** Roman Leischik

**Affiliations:** 1 Faculty of Health, School of Medicine, University Witten/Herdecke, Witten, Germany

Echocardiography is an informative method for examination of cardiac structures and not dispensable in examination of athletes. Dr Grazioli focused his paper on analysis of left and right ventricle and did it well. Right and left atrial dimensions ^1^^-^^3^ (and function ^3^^, ^^4^), aortic dimension ^5^^,^^6^ (and stiffness ^5^^, ^^7^) or pulmonary dimensions and function ^8^ were not focused in this initial article of the sports cardiology channel because  it is problematic to start with and to write a complete update with the burden of complete echocardiographic potential/possibilities. In one of his own papers he described alterations of aortic valve/pulmonary valve/mitral valve in athletes and aortic root dilatation or severe pulmonary stenosis ^9^.

Echocardiography was recommended for screening of athletes under different circumstances ^10^^, ^^13^ and particularly for follow up ^14^^, ^^15^ examinations. The possibilities of strain echocardiography were discussed in the past ^16^^, ^^17^ and we look forward to new. The global longitudinal strain has better reproducibility ^18^, the potential of radial strain seems to be limited in examination of training effects ^19^.  

 I recommend indexing of this article with the option to write an update with further possibilities and importance of echocardiography in the examinations of atrial function/valve and aortic function including 3D echocardiography and the further analysis of recreational  athletes ^20^ and importance of follow-up examinations ^21^^, ^^22^. The advantage of this sports cardiology channel is the possibility for updating papers with new literature and information and to actualize the growing knowledge in sports cardiology.

I have read this submission. I believe that I have an appropriate level of expertise to confirm that it is of an acceptable scientific standard.

## References

[1] D’AndreaADe CoratoGScarafileRRomanoSReiglerLMitaCAlloccaFLimongelliGGifantinoGLiccardoBCuomoSTagliamonteGCasoPCalbróR: Left atrial myocardial function in either physiological or pathological left ventricular hypertrophy: a two-dimensional speckle strain study. *Br J Sports Med*.2008;42(8) : 10.1136/bjsm.2007.041210696-702 10.1136/bjsm.2007.041210 Reference source 18070810

[2] D'AndreaARieglerLCocchiaRScarafileRSalernoGGravinoRGoliaEVrizOCitroRLimongelliGCalabròPDi SalvoGCasoPRussoMGBossoneECalabròR: Left atrial volume index in highly trained athletes. *Am Heart J*.2010;159(6) : 10.1016/j.ahj.2010.03.0361155-1161 10.1016/j.ahj.2010.03.036 Reference source 20569734

[3] D'AscenziFPellicciaANataliBMZacàVCameliMAlvinoFMalandrinoAPalmitestaPZorziACorradoDBonifaziMMondilloS: Morphological and Functional Adaptation of Left and Right Atria Induced by Training in Highly Trained Female Athletes. *Circ Cardiovasc Imaging*.2014;7(2) : 10.1161/CIRCIMAGING.113.001345222-229 10.1161/CIRCIMAGING.113.001345 Reference source 24470314

[4] LeischikRLittwitzHDworrakBGargPZhuMSahnDJHorlitzM: Evaluation of Left Atrial Mechanics: Function, History, Novel Techniques, Advantages, and Pitfalls. *Biomed Res Int*.2015;2015: 10.1155/2015/765921 10.1155/2015/765921 Reference source PMC450838526236735

[5] D'AndreaACocchiaRRieglerLSalernoGScarafileRCitroRVrizOLimongelliGDi SalvoGCasoPBossoneECalabròRRussoMG: Aortic stiffness and distensibility in top-level athletes. *J Am Soc Echocardiogr*.2012;25(5) : 10.1016/j.echo.2011.12.021561-567 10.1016/j.echo.2011.12.021 Reference source 22245050

[6] D'AndreaACocchiaRRieglerLScarafileRSalernoGGravinoRVrizOCitroRLimongelliGDi SalvoGCuomoSCasoPRussoMGCalabròRBossoneE: Aortic root dimensions in elite athletes. *Am J Cardiol*.2010;105(11) : 10.1016/j.amjcard.2010.01.0281629-1634 10.1016/j.amjcard.2010.01.028 Reference source 20494674

[7] NistriSGrande-AllenJNoaleMBassoCSivieroPMaggiSCrepaldiGThieneG: Aortic elasticity and size in bicuspid aortic valve syndrome. *Eur Heart J*.2008;29(4) : 10.1093/eurheartj/ehm528472-479 10.1093/eurheartj/ehm528 Reference source 18096569

[8] BossoneED'AndreaAD'AltoMCitroRArgientoPFerraraFCittadiniARubenfireMNaeijeR: Echocardiography in pulmonary arterial hypertension: from diagnosis to prognosis. *J Am Soc Echocardiogr*.2013;26(1) : 10.1016/j.echo.2012.10.0091-14 10.1016/j.echo.2012.10.009 Reference source 23140849

[9] GrazioliGMerinoBMontserratSVidalBAzquetaMPareCSarquella-BrugadaGYangüasXPiRTilLEscodaJBrugadaJSitgedM: Usefulness of Echocardiography in Preparticipation Screening of Competitive Athletes. *Rev Esp Cardiol (Engl Ed)*.2014;67(9) : 10.1016/j.rec.2013.11.023701-715 10.1016/j.rec.2013.11.023 Reference source 25172065

[10] YimES: Aortic root disease in athletes: aortic root dilation, anomalous coronary artery, bicuspid aortic valve, and Marfan's syndrome. *Sports Med*.2013;43(8) : 10.1007/s40279-013-0057-6721-732 10.1007/s40279-013-0057-6 Reference source 23674060

[11] YimESBasilicoFCorradoG: Early screening for cardiovascular abnormalities with preparticipation echocardiography: utility of focused physician-operated echocardiography in preparticipation screening of athletes. *J Ultrasound Med*.2014;33(2) : 10.7863/ultra.33.2.307307-313 10.7863/ultra.33.2.307 Reference source 24449734

[12] CalòLSperandiiFMartinoAGuerraECavarrettaEQuarantaFRuvoEdSciarraLParisiANigroASpataroAPigozziF: Echocardiographic findings in 2261 peri-pubertal athletes with or without inverted T waves at electrocardiogram. *Heart*.2015;101(3) : 10.1136/heartjnl-2014-306110193-200 10.1136/heartjnl-2014-306110 Reference source 25381327

[13] PaterickTEGordonTSpiegelD: Echocardiography: Profiling of the Athlete's Heart. *J Am Soc Echocardiogr*.2014;27(9) : 10.1016/j.echo.2014.06.008940-948 10.1016/j.echo.2014.06.008 Reference source 25042411

[14] LeischikRDworrakBFoshagPStraussMSpelsbergNLittwitzHHorlitzM: Pre-Participation and Follow-Up Screening of Athletes for Endurance Sport. *J Clin Med Res*.2015;7(6) : 10.14740/jocmr2129w385-392 10.14740/jocmr2129w 25883700PMC4394910

[15] LeischikR: Endurance Sport and Cardiac Injury. *Kardiol Pol*.2014;72(7) : 10.5603/KP.a2014.0089587-597 10.5603/KP.a2014.0089 Reference source 24846352

[16] D'AndreaACocchiaRRieglerLScarafileRSalernoGGravinoRGoliaEPezzulloECitroRLimongelliGPacileoGCuomoSCasoPRussoMGBossoneECalabròR: Left ventricular myocardial velocities and deformation indexes in top-level athletes. *J Am Soc Echocardiogr*.2010;23(12) : 10.1016/j.echo.2010.09.0201281-1288 10.1016/j.echo.2010.09.020 Reference source 21111925

[17] CaselliSMontesantiDAutoreCDi PaoloFMPisicchioCSqueoMRMusumeciBSpataroAPandianNGPellicciaA: Patterns of left ventricular longitudinal strain and strain rate in Olympic athletes. *J Am Soc Echocardiogr*.2015;28(2) : 10.1016/j.echo.2014.10.010245-253 10.1016/j.echo.2014.10.010 Reference source 25455545

[18] LeischikRDworrakBHenselK: Intraobserver and interobserver reproducibility for radial, circumferential and longitudinal strain echocardiography. *Open Cardiovasc Med J*.2014;8: 10.2174/1874192401408010102102-109 10.2174/1874192401408010102 25356089PMC4211138

[19] VitarelliACapotostoLPlacanicaGCaranciFPergoliniMZardoFMartinoFDe ChiaraSVitarelliM: Comprehensive assessment of biventricular function and aortic stiffness in athletes with different forms of training by three-dimensional echocardiography and strain imaging. *Eur Heart J Cardiovasc Imaging*.2013;14(10) : 10.1093/ehjci/jes2981010-1020 10.1093/ehjci/jes298 23299399

[20] LeischikRSpelsbergN: Endurance sport and "cardiac injury": a prospective study of recreational ironman athletes. *Int J Environ Res Public Heath*.2014;11(9) : 10.3390/ijerph1109090829082-9100 10.3390/ijerph110909082 Reference source 25192145PMC4199008

[21] LeischikRDworrakB: Ugly duckling or Nosferatu? Cardiac injury in endurance sport – screening recommendations. *Eur Rev Med Pharmacol Sci*.2014;18(21) :3274-3290 Reference source 25487940

[22] DemirelliSSamCTErmisEDegirmenciHSenIArisoyAArslanKDumanHSimsekZ: Long‐Term Cardiac Remodeling in Elite Athletes: Assessment by Tissue Doppler and Speckle Tracking Echocardiography. *Echocardiography*.2014; 10.1111/echo.12860 10.1111/echo.12860 25471592

# Peer review: Referee response for version 1

**DOI:** 10.5256/f1000research.7082.r9496

**Published:** 2015-07-20

**Authors:** Flavio D'Ascenzi

**Affiliations:** 1 Department of Medical Biotechnologies, University of Siena, Siena, Italy

The paper by Grazioli and colleagues provides a comprehensive review on the current knowledge and the clinical implications of echocardiography when applied to the evaluation of athlete’s heart in the context of pre-participation screening. The paper is currently focused on right and left ventricles, is well written, and analyses not only standard echocardiography, but also the novel echocardiographic techniques applied to athlete’s heart, with a specific focus on the differential diagnosis between exercise-induced physiological remodelling and the diseases most commonly cause of sudden cardiac death.

Major revisions

Although the clinical implications of biatrial analysis has to be understood, in my opinion a brief paragraph on the analysis of biatrial morphology and function could be potentially of interest, also considering the some authors have demonstrated its utility in the differential diagnosis between athlete’s heart and cardiomyopathies.

An interesting point of using echocardiography in the context of a screening has not been analysed. Although some authors debate about the utility and the costs of echocardiography, in my opinion the fundamental question of the application of echocardiography to the pre-participation screening in athletes is WHEN performing this exam. Considering that a frequent echocardiographic evaluation is not feasible in low-risk populations and dramatically increases the cost in absence of real clinical advantages, one has to suppose that one or two exams can be performed during the career of a non-professional athlete. Although some congenital defects can be detected in the early phase of the life of an individual, some of the most common cause of sudden cardiac death are the genetic myocardial diseases, such as HCM, DCM, and ARVC, all known to have a delayed phenotype expression.  Thus, performing an echocardiogram at the beginning of the athletic career of a child could detect congenital defects, but could be misleading in excluding the cardiomyopathies most commonly associated with sudden cardiac death, with a typical expression in the II-III (or even IV) decades of life of a young athlete. I think that the authors should analyse this clinical question and should provide a personal opinion based on the current available evidences and on the experience of their Center.

Minor revisions

In table 1 I think that “aortic coartation” should be included in the section “suprasternal view”.

Table 2: exercise has not proven to be a determinant of a physiological dilation of the aorta; conversely, it has been demonstrated that aortic dimensions are comparable with those of the general population. Accordingly, I suggest removing “mild aortic dilation” from table 2, section “athlete’s heart”.

Atherosclerotic coronary artery disease cannot be detected by echocardiography, although indirect signs such as left ventricular wall motion abnormalities can suggest its presence. However,  considering the signs are uncommonly observed in athletes, I suggest to remove “Atherosclerotic coronary artery disease” from table 2, section “sudden cardiac death”.

Left ventricular non compaction: “it might be another expression of cardiac adaptation to increased preload and afterload influenced by genetic and ethnical factor”: ref. #51 is not correct because does refer to an hypothesis rather than an explanation of the phenomenon. I suggest to cite the article by Gati S. *et al *(Circulation 2014) and the work by D’Ascenzi F. *et al* (Int J Cardiol 2015) both explaining the physiological determinants of benign hypertrabeculation in the athletes.

Table 3: the definition “competitive athletes, high static and dynamic component endurance training, > 10 hours/week” is not clear: which is the opinion of the authors? Although it is clear that echocardiography is not recommended in subjects engaged in recreational sports, which are the athletes to be evaluated by echocardiography? All the competitive athletes? Athletes with abnormal findings at resting ECG or at history/physical examination? Athletes engaged in high-volume training programs?...Please specify.

Table 4

At the present time, LV circumferential and radial strains cannot be definitively suggested to distinguish between cardiomyopathies and athlete’s heart. LV longitudinal strain is highly sensitive for myocardial disorders and more reproducible than circumferential and radial strains ((Feigenbaum H, Circ J 2012). Because of the LV shortens from base to apex in systole, the fixed short-axis tracking that is required for circumferential and radial strain is more difficult than longitudinal tracking. Particularly, radial strain is intrinsically more vulnerable to measurement variability because strain assessed in these orientations represents an aggregate measure of endocardial, mesocardial and epicardial tissue motion, encompassing both myocardial fiber shortening and global LV torsion in systole. The best inter- and intra-observed reproducibility is observed for LV longitudinal strain and particularly data based on measurements of LV radial strain should be interpreted with caution (Cheng S, J Am Soc Echocardiogr 2013). A limited reliability has been observed also for LV circumferential strain measurements. A possible explanation is that circumferential deformation twist can hardly be assessed in the same view, because the base descends to the apex. This movement makes the correct measurement in the short axis view impossible in two different time-points or even in two different days.

Finally, LV longitudinal strain measurements are known to be more robust than radial ones (Mor-Avi, Eur J Echocardiogr 2011). According to these considerations and to the fact that LV longitudinal strain is the only form of strain that is used routinely (Feigenbaum H, Circ J 2012), I suggest to remove both radial and circumferential strains from table 4 and to stress in the text that future studies are needed to evaluate whether these deformation parameters could be used in the clinical practice.

“motion abnormalities” -> please correct with “wall motion abnormalities”

Table 4: according to the current available data on deformation analysis applied to the athlete’s heart, I suggest to describe LV GLS at rest as “normal” and not “normal or slightly reduced”.

I have read this submission. I believe that I have an appropriate level of expertise to confirm that it is of an acceptable scientific standard, however I have significant reservations, as outlined above.

# Peer review: Referee response for version 1

**DOI:** 10.5256/f1000research.7082.r9040

**Published:** 2015-06-26

**Authors:** Andreas Müssigbrodt

**Affiliations:** 1 Department of Electrophysiology, University of Leipzig, Leipzig, Germany

This review summarizes some important aspects of echocardiography as a screening tool for the screening of athletes and should therefore be of interest for sports physicians and cardiologists. The potential advantages of this method and the technical aspects are well described.

I would nevertheless recommend to mention potential problems too, mainly overdiagnosis and misdiagnosis with important consequences for the career of athletes and additional costs.  In order to prevent overdiagnosis and misdiagnosis, intensive training of sports cardiologists seems to be of utmost importance.

Furthermore, as research in this area often focuses on young, competitive athletes, I would recommend to discuss the potential value of echocardiography for older, non-competitive, recreational athletes.

I recommend indexing after minor revision.

I have read this submission. I believe that I have an appropriate level of expertise to confirm that it is of an acceptable scientific standard.
